# Parallel workflow tools to facilitate human brain MRI post-processing

**DOI:** 10.3389/fnins.2015.00171

**Published:** 2015-05-13

**Authors:** Zaixu Cui, Chenxi Zhao, Gaolang Gong

**Affiliations:** State Key Laboratory of Cognitive Neuroscience and Learning and IDG/McGovern Institute for Brain Research, Beijing Normal UniversityBeijing, China

**Keywords:** human brain, workflow tools, parallelization, multi-modal MRI, image post-processing

## Abstract

Multi-modal magnetic resonance imaging (MRI) techniques are widely applied in human brain studies. To obtain specific brain measures of interest from MRI datasets, a number of complex image post-processing steps are typically required. Parallel workflow tools have recently been developed, concatenating individual processing steps and enabling fully automated processing of raw MRI data to obtain the final results. These workflow tools are also designed to make optimal use of available computational resources and to support the parallel processing of different subjects or of independent processing steps for a single subject. Automated, parallel MRI post-processing tools can greatly facilitate relevant brain investigations and are being increasingly applied. In this review, we briefly summarize these parallel workflow tools and discuss relevant issues.

## Introduction

Over the past two decades, magnetic resonance imaging (MRI) techniques have been increasingly applied in brain research, and particularly research on the human brain, due to their non-invasive nature and outstanding spatial resolution for measuring brain structure and function. These techniques typically generate large-scale imaging datasets. To obtain specific brain measures of interest from an acquired MRI dataset, complex image post-processing steps are required.

A number of publicly available software packages have been developed to process brain MRI data, such as FMRIB Software Library (FSL) (Smith et al., 2004), Statistical Parametric Mapping (SPM) (Ashburner, 2012), FreeSurfer (Fischl, 2012), Analysis of Functional NeuroImages (AFNI) (Cox, 1996), BrainSuite (Shattuck and Leahy, 2002), Camino (Cook et al., 2006), CONN (Whitfield-Gabrieli and Nieto-Castanon, 2012) and Diffusion Toolkit (Wang et al., 2007). These packages provide **processing modules** and interfaces to comprehensively analyze multi-modal brain MRI data. To use these packages, end users must correctly understand each module and manually combine the appropriate modules for a particular purpose. In most packages, end users must also process each step or dataset separately, which is a sub-optimal approach for two reasons. First, understanding the various modules is difficult, particularly for investigators without computational backgrounds. Second, the use of these modules typically involves a number of manual operations, which increases the probability of processing errors due to user oversight. In contrast, automated workflow tools that allow user-operated processing steps to be concatenated enable fully automated processing of raw MRI data.

KEY CONCEPT 1 **Processing modules**

A function/script to achieve a specific processing purpose, e.g., image segmentation or registration.

Human neuroimaging studies typically require a large number of subjects. Thus, the same post-processing procedures are executed across different datasets. Certain workflow tools, such as those embedded in SPM and AFNI, can automatically and sequentially process different individual datasets. The independent post-processing steps for each individual dataset are also performed sequentially. This sequential processing pattern may not fully optimize available computational resources in a system (e.g., a multi-core desktop/server, a local distributed computing cluster or a high-performance computing platform), resulting in an unnecessarily long computational time. Computational time is becoming increasingly important due to the rapidly increasing sample size of human brain MRI studies. To address this issue, MRI data post-processing tasks across different individuals or within one individual can be parallelized by assigning independent post-processing jobs to different computing cores. Because the majority of personal computers and workstations possess multi-core systems and given that many research centers are now equipped with local distributed computing clusters or high-performance computing platforms, the adoption of workflow tools that permit the automatic **parallelization** of post-processing steps and optimal use of available computational resources is now possible.

KEY CONCEPT 2 **Parallelization**

A mode in which processing jobs without dependency run at the same time, with each job occupying a computing core.

A few parallel workflow packages for brain **MRI post-processing** have been developed. These tools can greatly facilitate relevant human brain MRI investigations and have attracted much attention in the research community. In this mini-review, we aim to provide an overview of these tools for human brain MRI and to discuss relevant issues for potential users and developers.

KEY CONCEPT 3 **MRI post-processing**

The computing/processing of raw images from multi-modal MRI techniques to obtain specific brain measures of interest.

## Available parallel workflow tools for multi-modal MRI post-processing

**Figure 1:**
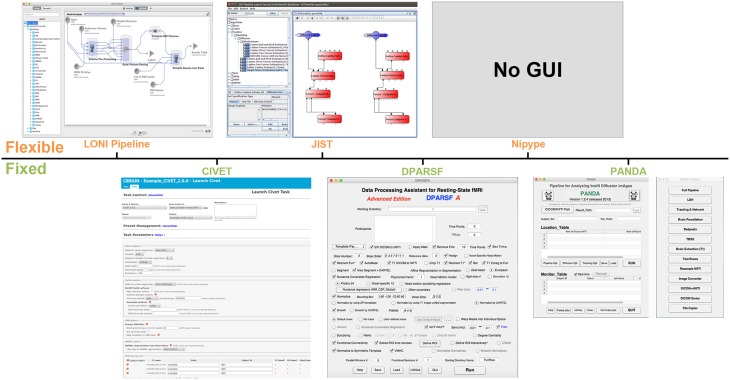
**Graphical user interface (GUI) snapshots for the example workflow tools.** LONI Pipeline, CIVET, JIST, DPARSF, Nipype, and PANDA are included.

In general, there are two categories of available parallel workflow tools for human brain MRI data processing (Table 1, Figure 1). One is **flexible workflow tools** that provide rich environments and allow users to customize automated workflows for any purpose by linking either modules from predefined libraries or in-house modules, such as Laboratory of Neuro Imaging (LONI) Pipeline (Rex et al., 2003; Dinov et al., 2009, 2010), Java Image Science Toolkit (JIST) (Lucas et al., 2010; Li et al., 2012) and Nipype (Gorgolewski et al., 2011). The other category is **fixed workflow tools** that provide a completely established data processing workflow for a particular purpose/dataset, such as CIVET (Ad-Dab'bagh et al., 2006), Pipeline for Analyzing braiN Diffusion imAges (PANDA) (Cui et al., 2013), and Data Processing Assistant for Resting-State fMRI (DPARSF) (Yan and Zang, 2010).

KEY CONCEPT 4 **Flexible workflow tools**

An environment that provides the ability to encapsulate modules from predefined libraries to create a completely automatic workflow.

KEY CONCEPT 5 **Fixed workflow tool**

A software package that concatenates a series of processing modules according to the dependency between the modules, allowing for fully automated processing, from the raw data to final outputs.

**Table 1:** **A comparison of example workflow tools for brain MRI post-processing**.

	**GUI**	**Platforms**	**Local parallel**	**Distributed computing^a^**	**Cloud storage**	**API**	**Specific to brain MRI**	**License**	**First release (year)**
LONI Pipeline	Yes	M, U, W	Yes	SGE, PBS, LSF, GridWay	Yes	XML Shell	No	LONI software license^b^	2003
JIST	Yes	M, U, W	Yes	SGE, PBS	No	Java	Yes	LGPL	2009
Nipype	No	M, U	Yes	SGE, PBS, HTCondor, LSF, SLURM	No	Python	Yes	BSD	2011
CIVET	Yes	M, U	Yes	SGE, PBS, Grid/Cloud	Yes	Shell	Yes	Work in progress^c^	2006
DPARSF	Yes	M, U, W	Yes	Matlabpool	No	Matlab	Yes	GPL	2009
PANDA	Yes	M, U	Yes	SGE, PBS	No	Matlab	Yes	GPL	2012

M, Mac; U, UNIX; W, Windows.

GUI, Graphical User Interface; SGE, Sun Grid Engine; PBS, Portable Batch System; LSF, Load Sharing Facility; SLURM, Simple Linux Utility for Resource Management; HTCondor, High-ThoughPut Condor; API, Application Programming Interface; XML, eXtensible Markup Language.

LGPL, GNU Lesser General Public License; BSD, Berkeley Software Distribution; GPL, GNU General Public License.

a PSOM, PBS, SGE, LSF (http://www-03.ibm.com/systems/platformcomputing/products/lsf/), SLURM (http://slurm.schedmd.com/), and HTCondor (http://research.cs.wisc.edu/htcondor/) are distributed resource management systems for job scheduling on distributed computing systems. They can allocate the computational resources according to the demands automatically. However, they are typically designed for local distributed computing systems. In contrast, GridWay (http://www.gridway.org/) can provide access for these in-house systems to grid infrastructures and cloud resources. Matlabpool is specific to Matlab, and can distribute Matlab scripts to multi-cores on a single computer or a distributed computing system. Similarly, the PSOM (https://www.nitrc.org/projects/psom/) is a framework that can implement pipelines in Matlab or Octave and distribute jobs to distributed resource management systems by calling PBS, SGE, and so on.

b http://loni.usc.edu/Software/license.php.

c According to the author's inquiring, the CIVET developers are currently working on the licensing issue.

## Flexible workflow tools

Well-designed flexible workflow packages for multi-modal MRI post-processing allow access to appropriate modules from existing software, such as FSL, FreeSurfer, SPM and AFNI, to construct a customized analysis. For example, LONI Pipeline and JIST provide a user-friendly graphical user interface (GUI) to let users create a complete neuroimaging analysis workflow, from raw imaging data to quantitative results ready for statistical analysis. To construct a workflow in the LONI Pipeline or JIST environment, users need to drag appropriate modules from the existing library, define the dependencies between these modules, and set the parameters for each module. Nipype, which is based on Python and which lacks a GUI, encapsulates processing modules of existing neuroimaging software as Python objects. These objects can be easily linked and executed as an automated workflow. In addition to customized workflows, akin to fixed packages, flexible packages provide certain completely established workflows, such as the tensor-based morphometry workflow in LONI Pipeline (Dinov et al., 2010), the cortical reconstruction using implicit surface evolution workflow in JIST (Lucas et al., 2010), and the diffusion data analysis workflow based on Camino in Nipype (http://nipy.sourceforge.net/nipype/interfaces/generated/nipype.workflows.dmri.camino.diffusion.html). To accelerate the data processing speed, these flexible packages all support parallel computing across multi-cores on a single computer or across multi-computers in a distributed computing cluster.

A flexible parallel workflow tool typically includes (1) a predefined library, (2) a workflow construction framework, (3) validation and quality control, (4) module creation, and (5) computational parallelization. In particular, a library encapsulating modules from existing neuroimaging software is first needed. These modules should be designed to allow for setting input, output, and parameter specifications, among other settings. The framework/protocol for connecting different modules in terms of between-module dependencies should be regularized. As manual setup of the workflow by users may lead to errors, automated validation that monitors the existence of input files, the consistency of data types, parameter matches, and protocol correctness is desired. Additionally, quality control, e.g., through visual inspection of the interim results, is also of great importance because this type of workflow processing is fully automated and nontransparent to users. A module creation framework/protocol that permits users to create their own modules is a plus because the modules in the predefined library may not meet the requirements of a particular analysis. Finally, implementing computational parallelization of independent jobs within the workflow is highly preferred to optimize the computational efficiency. We will illustrate these points using LONI Pipeline as an example.

### Predefined library

As an environment for constructing an integrated workflow with heterogeneous neuroimaging toolboxes, LONI Pipeline has a library of various modules based on popular MRI packages, such as AFNI, SPM, FSL, FreeSurfer, and Diffusion Toolkit. A very user-friendly and uniform interface has been designed for various modules.

### Workflow construction framework

LONI Pipeline provides a canvas for creating and revising a workflow in the main GUI. To construct a workflow, users only need to drag the appropriate modules from the library, to link the output of one module to the input of another module, and to define input/output files and parameters. Additionally, LONI Pipeline can automatically determine the most appropriate analysis protocol, select corresponding modules, and generate a valid graphical workflow according to the workflow description and a set of user-specified keywords.

### Validation and quality control

As the workflow is manually constructed, errors are possible. LONI Pipeline supports automatic validation of the consistency of the data types, of parameter matches, and of protocol correctness in advance of executing any workflow. For quality control, users can view the interim results of each module by clicking the icon for each module on the canvas during the execution of the workflow in the LONI Pipeline environment.

### Module creation

LONI Pipeline permits users to create their own modules in the case that the existing modules in the library cannot meet their requirements. The module description typically includes general information (e.g., name, package, authorship, citation), parameter specification (e.g., parameter/file type, dependencies), and executable information (e.g., program location, grid-specific variables). Users can define the description using a user-friendly GUI for module definition. Additionally, several ways to automatically create modules are supplied. Given this feature, LONI Pipeline can therefore also be applied to construct workflows for non-MRI related processing (e.g., genetic analysis).

### Computational parallelization

LONI Pipeline can execute thousands of simultaneous and independent jobs on a multi-core system, a distributed cluster, or a gird/cloud computing system using job scheduling tools such as Sun Grid Engine (SGE), Portable Batch System (PBS), Load Sharing Facility (LSF), and GridWay.

## Fixed workflow tools

Fixed parallel workflow tools have been developed for particular types of human brain MRI post-processing for which a fully automated processing workflow is completely established and ready for use. For example, the CIVET pipeline tool was developed to facilitate cortical morphological analysis (Ad-Dab'bagh et al., 2006). In CIVET, the raw T1-weighted images are the input, and cortical measures, such as thickness and surface area, are the outputs after implementing a number of image processing steps, e.g., brain tissue segmentation, spatial normalization, surface extraction, and surface registration. It has been recently embedded in the Canadian Brain Imaging Research Platform (CBRAIN) system, which is a web-based neuroimaging research platform designed for computationally intensive analyses using high-performance computing clusters/servers around the world (Sherif et al., 2014). Another example is PANDA, which is a diffusion MRI post-processing pipeline tool (Cui et al., 2013). PANDA specifically integrates several publicly available packages' modules (e.g., FSL) and in-house modules to accomplish all required pre-processing steps for diffusion MRI. The final outputs include brain diffusion metrics and white-matter networks ready for statistical analysis. To post-process resting-state functional MRI data, an automated workflow package called DPARSF [part of toolbox for Data Processing and Analysis of Brain Imaging (DPABI) (http://rfmri.org/dpabi)] has been developed (Yan and Zang, 2010). DPARSF can yield various brain functional metrics for statistical analysis, such as the regional homogeneity (Zang et al., 2004) and amplitude of low-frequency fluctuations (Zang et al., 2007), by integrating various modules from SPM and RESting-state fMRI data analysis Toolkit (REST) (Song et al., 2011).

CIVET, PANDA, and DPARSF do not require users to customize the workflow by selecting modules or defining dependencies. In fact, once the user inputs the raw MRI datasets and selects the post-processing parameter configurations, these tools fully automate all post-processing steps for all datasets. Additionally, these tools all enable parallel computing on a multi-core computer. CIVET and PANDA can also support a distributed computing cluster or a high-performance computing platform.

**Figure 2:**
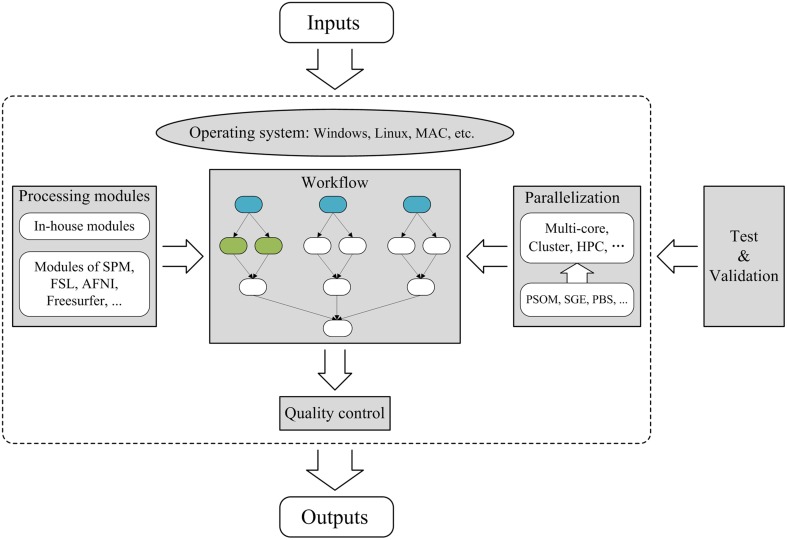
**Framework for the construction of a parallel workflow tool for brain MRI post-processing.** The sections with gray backgrounds represent important aspects of the construction of a parallel workflow tool. In the workflow section, the three blue nodes represent the same post-processing jobs from three different subjects and are therefore independent. The two green nodes indicate two independent post-processing jobs for the same subject. The arrows denote dependencies; for example, A→B indicates that B cannot start until A is complete. Thus, independent jobs can be parallelized to maximize the use of available computing resources. HPC, High-Performance Computing; SGE, Sun Grid Engine; PBS, Portable Batch System.

To construct a fixed workflow tool for brain MRI post-processing, several factors must be considered: (1) the operating environment and processing modules, (2) workflow design, (3) parallelization, (4) quality control, and (5) testing and validation, as illustrated in Figure 2. Typically, a fixed workflow tool requires a combination of in-house modules and existing modules from publicly available packages (e.g., FSL, SPM). However, because certain publicly available packages are only compliant with specific operating systems (e.g., Windows, Linux, or MAC), a fixed workflow tool must first specify the operating system requirement. Next, the workflow must be designed according to acceptable standard protocols for relevant MRI post-processing procedures. Typically, the workflow comprises a number of interconnected or parallel jobs, each of which is an MRI post-processing unit. To achieve parallelization, specific tools [e.g., Pipeline System for Octave and Matlab (PSOM) (Bellec et al., 2012), SGE and PBS] for managing computing resources must be applied within the workflow to enable the execution of independent jobs in parallel. As for flexible workflow tools, quality control is also critical for a fixed workflow tool, and effective strategies for quality confirmation must be carefully designed within the tool. Finally, fixed workflow tools must be thoroughly tested and validated by various users to minimize MRI post-processing errors and to ensure that the GUI is as user friendly as possible. These aspects will be elaborated using PANDA as an example.

### Operating system and processing modules

To efficiently obtain various diffusion metrics and brain networks that are ready for statistical analysis, PANDA was designed to combine a number of in-house post-processing modules with existing modules from publicly available packages [e.g., FSL, Diffusion Toolkit and MRIcron (http://www.mccauslandcenter.sc.edu/mricro/mricron/)]. Because the FSL package is compatible only with UNIX-based (e.g., Linux or MAC) operating systems, PANDA was designed for a UNIX-based system.

### Workflow design

The processing workflow within PANDA follows the recommended practices for the post-processing of diffusion MRI images in the research community. The main procedure comprises three parts: (I) pre-processing, (II) production of diffusion metrics, and III) construction of brain networks. Part I includes the following steps: (1) converting DICOM files into NIfTI images, (2) estimating the brain mask, (3) cropping raw images to reduce the memory cost and accelerate processing in subsequent steps, (4) correcting for the eddy-current effect, (5) averaging multiple acquisitions, and (6) calculating diffusion metrics. Part II consists of normalizing and computing multi-level diffusion metrics that can be directly used for voxel-level, atlas-level and tract-based spatial statistics (TBSS) level statistical analysis. Part III tasks include defining network nodes (i.e., parcellating gray matter into multiple regions) and constructing brain networks using deterministic and probabilistic tractography, respectively. Overall, the entire workflow of PANDA comprises 176 post-processing jobs for a single diffusion MRI dataset.

### Parallelization

The post-processing jobs within PANDA are organized using PSOM. The dependencies between jobs are first defined. According to PSOM, all jobs that are independent of all other jobs can be executed in parallel, including post-processing jobs for different individuals and independent jobs for the same subject. The processing status of each subject can be viewed in the GUI, with each job being assigned a status of “wait,” “submitted,” “running,” “failed.” or “finished.”

### Quality control

In PANDA, a results folder named “quality control” is generated. Snapshot pictures of the gray-matter parcellation atlas and of the fractional anisotropy and T1 images in both native and standard spaces are saved to this folder. These pictures can be used to quickly confirm the quality of the signal-noise ratio of the raw image, the quality of the spatial normalization, and the quality of the gray-matter parcellation. For the construction of the brain network, snapshot pictures of the whole-brain white-matter-tract map, which is derived from whole-brain deterministic tractography, are also produced to confirm quality by visual inspection.

### Testing and validation

Image post-processing procedures within PANDA are carefully implemented, and frequently used parameters are set as the default. PANDA was thoroughly tested and validated by students and collaborators before its official release.

## Discussion

Flexible (e.g., LONI Pipeline, JIST, and Nipype) and fixed (e.g., CIVET, PANDA, and DPARSF) parallel workflow tools are both widely used in the neuroimaging field. These tools can substantially simplify human brain MRI post-processing and optimize available computational resources. The tool choice for a specific study depends on application domains, users' background/preference, and access to computational resources.

Using flexible workflow tools, users can create any desired workflow using available modules in the library together with user-generated modules. To establish an appropriate workflow in such a modular environment, users need to have a good understanding of each individual module as well as the entire workflow protocol for analysis. Once the protocol and the appropriate modules are determined, users can construct the desired pipelines through linking these modules, defining input/output files and parameters with uniform and easy-to-use interfaces. Certain predesigned workflows that can be directly applied for specific analyses are typically included, but these existing workflows are likely to be less comprehensive than a specific fixed workflow tool for similar analyses.

In contrast, fixed workflow tools typically implement comprehensive processing and yield a series of resultant outputs, but only for a particular MRI modality (e.g., structural MRI, diffusion MRI, or functional MRI). All processing steps are pre-included and pre-linked following widely accepted protocols in the research community, so users do not bear the burden of designing the workflow and selecting/building the modules. However, given the diversity of MRI modalities, more efforts are warranted to develop fixed but comprehensive parallel workflow tools for diverse purposes.

The majority of workflow tools are designed to allow for parallel computation with available computing resources (i.e., multi-core desktop, GPUs, local clusters, high-performance computing, and grid/cloud computing), and therefore can greatly accelerate data processing of rapidly increasing neuroimaging datasets. For example, both LONI Pipeline and PANDA can parallelize jobs on a multi-core system or a distributed cluster. LONI pipeline also supports a grid/cloud computing system. Particularly, there is a newly released tool for parallel pipeline analyses of fMRI data on GPUs, i.e., BROCCOLI (Eklund et al., 2014).

Several points, however, need to be emphasized for both users and potential developers. First, quality control is essential for automated workflow tools. In particular, once the entire post-processing procedure is complete, the processing quality must be verified prior to initiating subsequent procedures. In most parallel workflow tools (e.g., LONI Pipeline, JIST, Nipype, CIVET, DPARSF, and PANDA), a number of intermediate results/snapshots are provided for rapid manual checks. In flexible workflow tools, automatic validation of the workflows customized by users (e.g., correctness of the analysis protocol, input/output types, and format compatibility) is also important.

Second, workflow tools typically allow the user to modify or rerun the workflow if processing errors are indicated by the validation or quality-control procedure. In fact, in a flexible workflow tool such as LONI Pipeline, users can redesign the workflow. In addition, both flexible and fixed workflow tools (e.g., LONI Pipeline and PANDA) permit users to modify parameters in specific processing steps and rerun the workflow if errors are found during quality control. Using these features, investigators can therefore easily evaluate the effects of processing strategies or parameters on the final results by rerunning workflows with different parameters or structures.

Third, certain MRI post-processing steps remain controversial. For example, there are valid reasons for removing or retaining the global signal when pre-processing a resting-state fMRI dataset (Fox et al., 2009; Murphy et al., 2009). In such a case, fixed workflow tools should offer options to the user, rather than implementing only one solution. Furthermore, with additional research and further development in the field, human brain MRI post-processing practices will likely change. Thus, workflow tools must be constantly updated to remain consistent with currently recommended practices. Ongoing technical support and debugging/updating of tools should be provided, e.g., via online forums or mailing lists.

Finally, workflow tools are advantageous for replications and validations of scientific findings in the human brain MRI research community. In most cases, applying the entire analysis procedure in the exact same way as in a publication is difficult due to insufficient description of the method and the numerical instability across different computing platforms (Glatard et al., 2015). In contrast, flexible workflow tools, such as LONI Pipeline, provide a clear and complete record of the analysis protocol, processing modules, parameters, input/output and computing platform information. Similarly, fixed workflow tools (e.g., PANDA) typically save a configuration containing all of the parameters, input/output and computing platform information. Including these relevant information for the processing workflows in a publication is therefore highly encouraged, which can increase the reproducibility and transparency for both data processing and computing platform, ultimately enhancing the comparability of results between studies or datasets.

In summary, a number of flexible and fixed workflow tools exist for human brain MRI post-processing. These tools can greatly facilitate data processing, can save computational time and effort, and are being increasingly used. The application of these easy-to-use tools is therefore highly recommended for neuroscientists, psychologists, and clinical investigators, and particularly those with few computing and programming skills.

### Conflict of interest statement

The authors declare that the research was conducted in the absence of any commercial or financial relationships that could be construed as a potential conflict of interest.
